# Bringing Forth Generativities Within Relational Disquiet: An Interview With Karl Tomm

**DOI:** 10.1111/famp.70030

**Published:** 2025-03-18

**Authors:** Carla Guanaes‐Lorenzi, Mónica Sesma‐Vazquez, Joaquín Gaete Silva, Inés Sametband, Karl Tomm

**Affiliations:** ^1^ University of São Paulo São Paulo Brazil; ^2^ University of Calgary Calgary Alberta Canada; ^3^ Calgary Family Therapy Centre Calgary Alberta Canada; ^4^ Mount Royal University Calgary Alberta Canada

**Keywords:** bringforthism, family therapy, relational disquiet, therapeutic process

## Abstract

From August 16th to 20th of 2023, the Calgary Family Therapy Centre (CFTC) 50th Anniversary and Conference: Bringing Forth Generativities Within Relational Disquiet was held in Calgary, Canada. The theme of the conference was *relational disquiet,* a notion introduced at the CFTC to raise awareness that something is experienced as amiss, uneasy, restless, or a sense of unfairness when something is experienced as not right during the therapeutic process. In preparation for the conference, we interviewed Dr. Karl Tomm, founder and senior advisor of the Calgary Family Therapy Centre and a professor of Psychiatry at the Cumming School of Medicine, University of Calgary. In this article, we present Karl's interview, where he explains the notion of disquiet as a relational response to unwanted differences about hopes or desires emerging in our interactions with one another. Karl shares a clinical example of a possible transition from disquiet to generativity in a family therapy process. We offer some theoretical reflections on how Karl navigates disquieting interactions in family therapy conversations and discuss potential actions that family therapists can take to bring forth generativities from within relational disquiet.

## 1 Introduction

The Calgary Family Therapy Centre (CFTC) is a non‐profit organization that was founded in 1973 by Dr. Karl Tomm, MD, FRCPC, and other colleagues, as part of the Alberta Health Services (AHS). The Centre provided family therapy at no cost for families in Calgary, and also became a training facility for systemic family therapists, psychologists, social workers, and medical interns. Karl, a Professor of Psychiatry at the Cumming School of Medicine, University of Calgary, was the director of the CFTC for 50 years until August 2023. During the past half of a century, Karl made numerous contributions to the field of family therapy. Among these are the *Interventive Interviewing* article series (Tomm 1987, 1987b, 1988), one of which was recently celebrated among the 24 most influential papers in the history of the field (Pote & Tatar, 2019). In addition, the Reflexive Listening Exercise and the Internalized Other Interview as a therapeutic interventions (Tomm and Acton 2011; Tomm 2025), and the Interpersonal Patterns Scope (IPscope) family therapy framework (Tomm et al. 2014) are internationally well‐known.

At the CFTC, supervisors, clinical staff, and consultants possess extensive knowledge of the *Interpersonal Patterns Scope* (IPscope) framework. Many of them also integrate various contemporary family therapy ideas into their family therapy practice and supervision, including solution‐focused, collaborative, narrative, reflecting teams, and just therapy. Additionally, the majority of the supervisory team is Taos Institute associates who identify as social constructionists and collaborative‐dialogic practitioners.

Therapists use the IPscope framework to describe and analyze how family members may engage in and maintain certain relationships, described as interpersonal patterns (IP). According to Tomm et al. (2014),

Interpersonal Patterns are formally defined as repetitive or recurrent interactions between two or more persons distinguished by an observer (often a systemic therapist) that highlight the coupling between two classes of behaviors, attitudes, feelings, ideas, or beliefs and that tend to be mutually reinforcing. (19)

Thus, family therapists distinguish IPs to give visibility to the circularity and mutuality of interactions; therefore, focusing not on family members individually, but on the interactive space (Tomm 2014).

Tomm (1988, 2014) proposes a pragmatic typology organized into three classes of IPs. The first are Core Patterns that include: Pathologizing Interpersonal Patterns (PIPs), Wellness Interpersonal Patterns (WIPs), and Healing Interpersonal Patterns (HIPs). The second are Transitioning Patterns and include: Transforming Interpersonal Patterns (TIPs) and Deteriorating Interpersonal Patterns (DIPs). Finally, Social Patterns include: Social‐Cultural Interactional Patterns (SCIPs) and Social Network Interpersonal Patterns (SNIPs). Through this typology, Tomm et al. (2014) describe family relationships in their trajectory from pathologizing patterns to therapeutic and wellness‐generating patterns, including their relationship with social and cultural discourses and the community social network. The IPscope framework makes visible the permanent and dynamic movement between interpersonal patterns. Conversations that take place in therapy can promote transformation (TIPs) in repetitive and recurring interpersonal patterns in families (PIPs), favoring HIPs and WIPs to establish themselves (Gaete et al. n.d.; Gaete et al. 2020; Tomm 2014). In family therapy clinical practice and research, the IPscope has been used to generate a greater procedural understanding of the effects of therapeutic interventions and family therapy outcomes (Gaete et al. 2020; Tomm et al. 2014).

Some other contributions of Karl and his team at the CFTC are less known to the public. In recent years, the CFTC team incorporated the deliberate exploration of the generative potential of *relational disquiet* during post‐consultation debriefing conversations. This practice was started by the CFTC team as a way of debriefing after their weekly team consultations, also known as *screenings*. Exploring the generative potential of relational disquiet proved to be so fruitful to the team that clinical supervisors have now extended this process to almost all post‐session conversations. During these conversations, as a group they reflect and critically examine the therapeutic conversation and process, asking questions such as: Did you notice any moments of relational disquiet during the family therapy session? How do you/can we make sense of such disquieting moments? What do these moments tell us about the family's pain(s) or about what any of their members may be struggling with? What actions were taken to move the conversation and relationship forward? Which moments were perceived as sparkling or generative? Did we experience any therapeutic mistakes and/or openings for alternative developments? What could the therapist have done differently? What was generative about what the therapist and the family members did together? These questions help family therapists raise awareness of how they are using their therapeutic theories and models, what the effect of their actions is in the therapeutic process, and explore new directions for their therapeutic work.

Thus, the CFTC team nurtured a conversational space for generative dialogues about therapists' ideas and practices, termed research as daily practice (St. George et al. 2015). These dialogues took place in different settings: group processes, research as daily practice meetings, clinical supervision, and therapists' self‐reflection when they engage in documenting clinical records. Additionally, by welcoming local and international researchers to join research as daily practice activities and further develop potentially publishable research projects on relational disquiet, the CFTC team has gained a better understanding of the generative potential of relational disquiet within the therapeutic and training/clinical supervisory processes.

## 2 Exploring Generativity Within the Disquiet

The present interview was conducted by Dr. Carla Guanaes‐Lorenzi (onwards Carla), Associate Professor at the University of São Paulo (Brazil) while working as a visiting professor at the University of Calgary. For 6 months, Carla worked jointly with the CFTC team and, particularly, with Drs. Mónica Sesma‐Vazquez, Inés Sametband, and Joaquín Gaete‐Silva, co‐authors of this paper, in developing research projects aimed at better understanding how family therapists navigate relational disquiet in therapeutic sessions. This interview is part of this collaborative research initiative between Carla and the CFTC team and was digitally audio recorded, transcribed, and edited by the co‐authors, jointly with Karl Tomm. Once we edited the interview, as a group we planned a reflexive exercise that consisted of engaging in dialogues to discuss some of the key points that, as co‐authors, we considered could be the main contributions towards conceptualizing the notion of relational disquiet in family therapy daily practice. We first summarize and later discuss these key points.

In the first part of the interview, Karl explains why he and the CFTC team explored the generative potential of relational disquiet. He introduces us to his understanding of disquieting interactions, emphasizing why he considers these important, and invites family therapists to explore creative ways to respond to these unfamiliar and uneasy situations that often occur in therapeutic encounters. Then, Karl describes the story of Colleen, a client with whom he worked in family therapy many years ago. Karl had Colleen's prior permission to present part of her story as an example of generativity in family therapy at the CFTC's 50th Anniversary Conference, and later contacted her to ask for her permission to publish this interview in *Family Process*. Since her story is a major part of the interview itself, and to ensure she would be able to provide informed consent for the publication of her story as part of the interview, Karl sent her the first draft of this article. Colleen suggested fictional names, made comments, and edited her story so we could move forward and protect her confidentiality, based on a relational and dialogic ethical stance. This clinical example was an invitation for therapists to resist the temptation of avoiding disquieting moments in conversation, and to rather feel the excitement that can come as a result of a therapist's persistent inquiry for better ways to attune to the family demands and relational pain. We hope readers appreciate this interview as much as we did, and that it raises questions that can help us keep reflecting about the complexities of our work as family therapists, while also collecting resources to bring forth the best of the families with whom we work.

## 3 Interview

Carla: Thank you, Karl, for accepting this interview. My first question is: when did you start using the word *disquiet*?

Karl: I can't remember for sure. Let's say it was probably around 10 years ago.

Carla: Why the term *disquiet*? Do you remember how this word came to my mind?

Karl: I remember that in our post‐session discussions, after the screening interviews, the conversations would ramble; they would go on and on and on… And there wasn't a clear structure on how we wanted to engage in conversation in the post‐session. So, we wanted to be more disciplined and were concerned about trying to tighten things up a bit. So, we decided to focus on particular questions in the post session discussion. And, as you know, the questions that now we like to ask are: What are the main PIPs and HIPS? And, what are the sparkling moments of therapeutic influence? But we wanted to also open space to notice the negative aspects of what was happening, where things weren't going well, and to not ignore that. Sometimes, the resource‐oriented therapies tend to bias the positive too much. So, I suggested that we look at mistakes. The CFTC team was not comfortable with the idea of focusing on mistakes *[laughs]*. I find it useful to recognize mistakes, because you learn from them, right? However, we found that the language of relational disquiet suited our group of therapists better, and people were more comfortable with paying attention to what made us feel uncomfortable within the session. So, that would help us focus on whatever was happening in the interview that could have been dealt with differently. We had many conversations around it, that's how things evolved. Once this focus became part of our post‐session discussion, it became a major resource for learning: paying attention to those moments.

Carla: What difference focusing on relational disquiet brought to what you were doing before? You said that it turned out to be a major learning resource. What did you notice was different in the possibilities that this focus brings?

Karl: I think it brings the possibility to go into greater depth about the process. To me, the phenomenon of disquiet is an embodied phenomenon. As human beings, we are kind of resonating with each other. When people are feeling uncomfortable, that comes from somewhere. There is some perturbation that triggers that feeling. So, being able to notice it, and then track it, to try to determine its origins is a way to get into a deeper level of conversational understanding. That is what I thought was so enriching about it, that it added depth to the conversation. And we didn't have to agree on it, like people would have different experiences of disquiets. There wasn't a consensus on that. Those differences were useful differences.

Carla: When you think about the term *disquiet*, what does it mean to you?

Karl: It means that something is not right and something is wrong here. And, of course, it implies some kind of judgment. People's values are also involved. But overall, I would say that there is a sense of unfairness. Something is just not right. Sometimes it is very overt that people are in intense conflict, for example, when they are disqualifying each other, shouting at each other, blaming each other, and so forth. But sometimes it is more subtle; they interact through microaggressions. We can identify those within a bodily sensation, and once we become focused on them, then we can start figuring out why they are arising. Sometimes we do not figure it out ourselves, but we figure it out in conversation with other therapy team members.

Carla: Does this mean that sometimes you can feel the disquiet in the session and not necessarily understand what is going on, but later on, you make sense of it during a post‐session conversation?

Karl: Yes.

Carla: Do you think there is a difference between discomfort and disquiet?

Karl: I would say yes. I think discomfort often arises in situations that are generative. Though disquiet can be a source of generativity too. When people are at the edge of their capabilities and they are stepping beyond where they have been before, there is discomfort. People are not comfortable; they might be a little bit anxious. But it could be a good thing that they are stretching themselves to go beyond where they have been before. So, I think that is a dimension of discomfort, which I would not associate with disquiet. Disquiet to me is more negative, more grounded in negative emotions. Discomfort could be negative, of course, but could also be positive. When disquiet arises, there is a response to the disquiet and that response has constraints associated with it because people want to diminish the disquiet; there is an impulse to reduce it.

Carla: If we look at the dictionary (Oxford English Dictionary n.d.), we will find other words as possible synonyms, such as anxiety or worry. Do you think there is a difference between these words?

Karl: I would say so, in the sense that I think with disquiet there's a sense of unfairness, and injustice associated with that experience. People being nervous about public speaking could be an example of anxiety. They're feeling discomfort, but it is not necessarily negative, and it will not bring generativity itself in terms of generating more energy to do what may be difficult to do. In this context, I would connect disquiet more with the sense of unfairness and injustice, experiencing that something is wrong.

Carla: In the backgrounder that you wrote for the conference (Tomm 2022), you mentioned different sources of relational disquiet. Could you share some examples?

Karl: Well, in the work that we do, the main source comes from current relational disquiets that exist in the family system we are working with. The disquiet in the family system raises disquiet in the therapeutic system. Because the therapists are attentive to the disquiets, they try to find ways to enable movement out of that disquiet and hopefully create some generativity.

Carla: This relational disquiet in the family system is probably related to moments when therapists recognize an intense PIP. Would you agree?

Karl: Yes. It triggers disquiet in the therapeutic system. So, it begins in the family system, but it triggers disquiet in the therapy system. Those disquiets then become a point of potential clinical work.

Carla: Would you mind expanding on this?

Karl: Well, I mean, there could be disquiets just coming up in the therapy system itself. For example, where there is a difference in values between family and therapist. So, it doesn't have to arise just within the family. For instance, a therapist may want to get their agenda fulfilled. If they're pushing their agenda, there may be pushback from the family. So, the disquiet can emerge from the therapy system as well. Or it can arise between the family and the referral source. The referral source may feel that the family needs to go for therapy, but the family does not really want to go for therapy. But they concede to the power and influence of the referral source. That could be the source of disquiet. The original source of initiative to seek some therapy input could come from outside the family.

Carla: You constantly remind us that it is not only about hyper‐focusing on identifying or examining the disquiets, but also about the generative potential of disquiets.

Karl: Yes.

Carla: What are these generative potentials, and how can we notice the generative potentials of disquiet in a therapeutic session?

Karl: Well, when the emotion shifts from the negative feeling and tension to excitement, and you feel the excitement, you feel that something new is coming up, and then you know that things are moving in the right direction.

Carla: With the disquiet you feel an embodied negative feeling, but in this case, you are noticing some excitement, which is a positive feeling. So, mainly, we might notice an emotional shift.

Karl: Well, I think emotions are a larger base out of which cognitions can then arise. Cognitions could be congruent with emotions. But to me, the emotion is a way to ground it, to know what the meaning of the cognitions really is. When I have a sense that there is something happening here, in terms of new possibilities, or new distinctions being made, or new patterns emerging, the excitement that comes out of that. And it's collective, it's not an individual thing. Frequently, several people can resonate with that emotion when the therapeutic process is going well.

Carla: We have heard you use the word *attunement* sometimes; would the word attunement fit here?

Karl: Attuning? Yes.

Carla: There will be an attunement among the therapist and the family members. In this case, for example, would the family and therapist share this excitement?

Karl: Yeah.

Carla: Would you explicitly mention to the family that you have noticed a disquieting situation?

Karl: Oh, yes. I would not necessarily use the language of disquiet. But I would certainly engage in conversation about it.

Carla: Do you have an example of how you would do that?

Karl: Well, if you name the phenomenon, then you create a distance between yourself and the phenomenon named. For example, you could name it as conflict or harassing, or as a PIP pattern, or whatever. Giving the relational disquiet a name makes it possible to examine it and focus on it, and have a relationship with that situation you just named. So, to me, that is probably one of the first steps towards generativity. Even if the naming is intrapersonal, within your own thoughts, you are drawing a distinction. You probably implicitly name something when you distinguish it. If you then bring that distinction into the conversation, that adds even more potential with respect to separating the disquiet from the persons that are generating the disquiet. And that, to me, is the beginning of the capacity to work on it, to work with it [pause]. One of the themes that has come out of our conversations over this past year, at the research as daily practice seminars, is considering relational disquiet as a call for action. I really like that idea. Because if we can connect the phenomenon of disquiet with the need to take action and the need to do something about it, then we might go somewhere. I really liked that. This takes us to a better place, rather than simply trying to escape the disquiet and avoid the discomfort, which is our natural tendency. Often, we try to move away or hide it or cover it up somehow. But instead of doing that, moving forward into acting upon it helps to release its potential for generativity.

Carla: What are these actions that could be taken in a situation where there is relational disquiet? Naming it?

Karl: Well, even before naming it, we need to be open and willing to approach it, to go to it, to not go away from it, go to it. Thus, as you go to it, then it becomes possible to draw distinctions around it, to give it a name. And then we can find the language and explore its possibilities.

Carla: One concept that we are trying to explore further in our relational disquiet project is “welcoming” the disquiet. It seems that some members of the CFTC therapy team are referring to this first moment of not avoiding disquiet, and some of them mentioned the word “sitting in” [with the disquiet]. We were considering “welcoming disquiet” as one of the concepts that we could explore further.

Karl: I am a bit uncomfortable with welcoming disquiet per se, because it almost implies the desire to bring it forth or to create disquiet. And I do not want to do that. I would not want to create unnecessary disquiet. But if it exists, if it is already there, then welcoming a response to it makes a lot of sense to me. I want to approach it. To welcome it in the sense of recognizing its potential, yes. Sort of being willing to see it. There is something worth paying attention to. And then I might feel like I am doing my job as a therapist by approaching it and not avoiding it.

Carla: In the backgrounder (Tomm 2022), you mention that relational disquiet could often be a “major driver of creativity or important new developments” (p. 3). Do you have an example from your therapy practice in which we could understand better how the disquiet turned into a major source of creativity?

Karl: I can share a story about a young woman who came to see me in therapy. She began by asking me about the confidentiality limits and who could see my records and how secure are the records, and so forth. Immediately I thought that there was probably something legalistic involved in her situation. Clearly, she wanted me to keep her information confidential. So, I tried to answer her questions as best as I could. Then she asked me: “Who do you talk to about your clients?” and so forth. And I said: “Well, I don't know what you want to share with me but I can say that I will not share with anybody what you want to share with me until I discuss it with you first, that I can assure you. But I can't say that I won't talk to someone else. It depends on what it is.” I wanted her to feel safe enough to share what was troubling her, but I didn't want to box myself into a corner and promise not to talk with others. So, that was enough for her to tell me that she had tried to hurt her daughter. So, this woman, who I will refer to as Colleen, to not use my client's real name, told me that a few weeks earlier she had pushed her 4‐year‐old daughter, let's call her Millie, out the window of their second story apartment. Colleen explained that she had warned Millie many times to not lean out the window, but Millie kept going to the window and Colleen kept demanding she move away. Then, on one occasion, when Millie once again was leaning out the window, Colleen became so frustrated that she felt an impulse to push her out. She acted on that impulse by lifting her legs and Millie toppled out. She pushed the daughter out the window, and her daughter fell and was hurt. Colleen became extremely upset about what she had done. Then she took her daughter to her mother's place, and her mother insisted that she get some therapy. This is how she came in to see me. Then she said to me: “I don't want you to phone Child Protective Services because they apprehended my daughter before, and it took a whole year to get her back, and I don't want to go through that again”. So, I found myself in a very disquieting bind because legally, I am supposed to report this, but that would be doing the opposite of what she was asking for. So, I asked her many questions: “Where is your daughter now?” and she said “she is with my mother”. So, the child was with her grandmother. I asked: “Was she badly hurt?”. She said: “No. She was bruised but not seriously”. And I continued: “Well, is your daughter going to stay with her grandmother for now?”, and she said yes. Then I asked: “And will your mother be willing to be involved in the therapy so I can get some collateral information?”, and she said yes as well. Then I said: “Okay, under those circumstances, I can accept your request. For now, I am not phoning Child Protection Services”. So, given that Millie appeared to be okay by living in a safe place with her grandmother, I agreed to not call Child Protection Services ‘at this time’. What I was doing was putting the purpose of the law above the letter of the law. The purpose of the law is to keep the child safe and the letter of law says you should report this. So, I didn't report because she asked me not to report and I wanted to be sure that I could create a good therapeutic relationship with her. If I had reported her, I don't think there was any chance of having a therapeutic relationship with her. So, what I basically did was to create conditions to work with her in therapy. As things unfolded in therapy, Colleen disclosed a history of being abused herself by her father, and so forth. And further, the father of her daughter Millie had been violent with her too, which had made it difficult for her to bond with Millie as an infant. She needed substantial support to cope with her own sense of injustice. Thus, there is a lot of background history that had contributed to the abuse she enacted with her daughter at that moment. I decided to work with her over a long period of time and things turned out that the grandmother actually raised the daughter until she became an adolescent. Colleen was involved in her daughter's care, but at a safe distance. Millie often asked to move back home with her mother, but the extended family remained firm about the living arrangements. Things unfolded in very positive ways. Eventually, when Millie was old enough to understand, Colleen expressed regret and apologized to her daughter. Later on, Colleen went to law school and she specialized in working with families in situations of abuse. I phoned her just a few weeks ago to ask for permission to use her story in my presentation at the CFTC conference (Tomm 2023) and she said “Yes, by all means”. She also told me that a judge spontaneously thanked her for her work with the families. The judge told her: “You are doing excellent work for these families!”. Isn't that amazing? If I would have reported her, she would probably be charged with assault, or whatever, because she did have the intent to harm her daughter. That was on her mind. But this outcome of her doing work with other abusive families and that judge spontaneously acknowledging her work as ‘excellent’ is a fantastic generative outcome, right? In addition, when she became a lawyer, she actually raised a lawsuit against her father for his years of abuse when she was young. It was fascinating to me how, if you're willing to find a way to create space for generativity, good things can happen. I wonder whether, given her lived experience, Colleen might have become far more skilled in understanding and adjudicating complex abuse situations than most of us ever could. If I had reported her when I first met her, she may have faced criminal charges, and may never have gone to law school.

Carla: Wow!

Karl: Isn't that something?

Carla: Yeah, it is amazing!

Karl: I forgot to tell you that six months after I started working with her, I got a phone call from Child Protection Services. They wanted information about Colleen and I said, “Well, I am not at liberty to share information about her because I haven't had a chance to ask my client”. In this situation, the caller told me that they already knew about what happened. He said “Well, we know you're seeing her and we want you to continue to see her.” And so, the next time I saw my client, I told her that I got this phone call. She laughed and said: “Oh, I know what happened”. Then she explained that she got into an argument with her sister and her sister threatened to phone Child Protective Services.

Carla: This is a remarkable story. At what moment did you feel disquiet? And what were your actions to move forward with this?

Karl: There were lots of disquiets. There was the disquiet of her experiencing hatred of her daughter because of what she represented, because of the father, and her own history. And then there was the disquiet about me being in a situation where I was being asked to do something that I ethically and legally shouldn't do. I was trying to find a way to engage her in a therapeutic relationship by not rejecting her main request, which was to not phone Child Protection Services. However, I also wanted to somehow create the possibility that we could move forward therapeutically. By the way, I should have mentioned that I didn't do this alone. I had the support of a team; we worked together as a team in this case. They helped me manage my disquiet. I had discussed the situation with the team because I could not entirely trust my own judgment. I had to be careful in terms of the decisions I was making. Fortunately, I have a very supportive team. Eventually, things worked out in the long run. It was really quite amazing.

Carla: That is great!

Karl: I will share with you another relevant piece about this story. I first worked with Colleen about 40 years ago, but not long ago, when Millie, the daughter, became an adult and got married and had her own son, I saw them again when the son got to be about the same age that Millie was when the incident originally happened. There was some conflict between Colleen and her daughter about allowing Colleen to see her grandson. Understandably, Millie was a bit hesitant given that her son was about the same age as Millie was when the original incident took place. They came back to see me again for more therapy, and I could support them in talking through that sensitive situation. They eventually agreed that as long as Colleen continued taking her antidepressant medication and agreed not to talk to the grandson about abuse issues in the family, Millie was OK with Colleen spending time with him. So, they managed to work things out. It's Its very fascinating to have such long‐term contact with this family. Fascinating.

Carla: It really is. One therapist that I interviewed at the CFTC for our other project said that, for her, the disquiet could be like a “teacher” because it leaves a lesson. So, we added this question to our interview guide: if disquiet could be seen as a teacher, what would be its main lessons?

Karl: That is a nice metaphor.

Carla: Yeah, I like that. What have been your disquiet's main lessons?

Karl: Well, the main lessons? I think one would be that the disquiet could be the beginning of more trauma, injury, and damage. Or it could be the beginning of creativity and new developments that are therapeutic and healing. So, I would say that the disquiet could mark a bifurcation point. This is a moment of opportunity, potentially, for things to go this direction rather than that direction.

Carla: How could a family therapist be more aware of those bifurcation moments? You mentioned in your backgrounder (Tomm 2022) that it is important for therapists to “become more and more mindful of the distinctions we draw and eventually bring forth in our doings in therapy” (6). So, when you talk about this bifurcation point, how could a therapist be more aware of what they are bringing forth in their actions?

Karl: I think the way to become more aware of it is to perform a recursion on your reflections, to think about your thinking, and to notice the effects on you, especially when you draw a particular distinction.

Carla: Is it like exploring the inner voice of the therapist?

Karl: Yes… I mean, if you listen to your listening, and how listening in that way has effects on you, and how it influences where you go. For instance, you can listen in certain ways when you ignore disquiet and avoid it. You could listen for soothing possibilities to pour oil on the troubled waters, and so on. Or, you can listen in ways that take you to the wellsprings of the disquiet, in terms of the injustice or the unfairness, and then potentially deconstruct those injustices. So, we need to notice our noticing, or distinguish our distinctions to see what effects they are having on us in our conversations.

Carla: I like the idea of noticing our noticing. How could we invite others to be excited about disquiet as you did here at the CFTC?

Karl: Well, I can identify a turning point in our program. It was many years ago, when after a screening, we were in a post session discussion. We were having a good conversation. I did the actual interview, and it was pretty good. We were talking about what happened in the session that was really useful, and so on, and then towards the end of the conversation, I said: “I think I made a mistake in the interview, when I didn't respond to this…”. The group was kind of shocked because they had put me on a pedestal. They had the belief that everything that I do in therapy must be perfect. Then, I explained why I believed it was a mistake and what we could learn from it. We got very excited because we were learning together. I also got excited because they were excited. Someone else had the courage to say, “well, I wondered about when you said X… because it could have been a problem.” So, I became excited with my new learning. When others on the team saw my excitement, they felt safer to ask: “Oh, yeah. What about…?”. So, we all got very excited because people were then basically sharing their disquiets, right? And that was the beginning of our change in the process, in our program and in our culture at the CFTC. When I, as the director, was willing to talk openly about my mistakes and discuss them, that to me was very helpful. Because when I can do that, then it becomes easier for other people to do similarly, and also allow themselves to be more vulnerable, because they can see the potential for learning that can come out of noticing the disquiet and reflecting on the potential mistakes.

Carla: When I read the IPscope book (Tomm et al. 2014), I noticed that the word “disquiet” is not there. What would be different in the IPscope approach if the understanding of disquiet was included? In what ways could the understanding of relational disquiet enrich the IPscope?

Karl: I think it would thicken it, enrich it quite a bit. Which is consistent with what I said earlier, that when we go into the disquiet, we gain more depth. I think that is a very valuable contribution of drawing that distinction. When we wrote the book, we had the distinction, but we were not harvesting it yet, you know, the generativity from it, or the fullest generativity possible.

Carla: If you think in the IPscope diagrams (Tomm et al. 2014) explaining the movement or transitions among PIPs and HIPs, and so on, where would you locate the disquiet?

Karl: Well, it is on the PIPs. Every PIP has disquiets.

Carla: Would the disquiet be in the movement?

Karl: Well, yeah, this is where the relationship between the intrapersonal and interpersonal comes into play, because disquiet is first felt intrapersonally. It shows up as a bodily uneasiness.

Carla: As a response to…

Karl: Yes, very much a response to a process of interaction, and the process of interaction that generates a PIP. So, yes, there is disquiet in every PIP.

Carla: Yeah, okay! If you write a new book, your book on disquiets [*mentioning a previous conversation that took place at the CFTC, referring to the title of Fernando Pessoa's Poetry book*, “The Book of Disquiets”] (Pessoa 2015). What would be its main chapters?

Karl: What I think has not yet been done, and I was hoping that could start at the CFTC conference, is starting a taxonomy of different generativities that could arise from entering into and working with disquiet. As an example, there are the bifurcations I mentioned earlier. *Bifurcation questioning* could be one example of a category of generativity. But I think there could be many categories and different kinds of generativities that could arise from disquiet. That analysis hasn't yet been done. And we haven't yet had those conversations as a team. Perhaps this could be a topic for our *Research as Daily Practice* monthly meetings group!

Carla: Can you explain to us a little bit more what do you mean by bifurcation questioning?

Karl: Well, maybe I could back up and give a bit of theoretical background. The term bifurcation comes from Ilya Prigogine and Isabelle Stengers's work in analyzing complex systems in their evolution, their theory of dissipative structures (Prigogine and Stengers 1984). In that work, what they found was that if a system is evolving and comes to a bifurcation point where, if the system evolves in one direction as opposed to the other direction, they end up in very different places. So, those bifurcation points are very important points when the system is evolving. And, in our understanding of ourselves and our relationships, we come to many bifurcation points. Thus, if a therapist can bring forth those bifurcation points, the therapist then invites people to recognize the alternatives and recognize themselves as having a choice between those alternatives, which is empowering for them. It opens space for them to have some agency, which is such a wonderful experience, to realize that you do have a choice, and you can make a choice for something being better, or follow your values and beliefs in terms of the direction that you're privileging in the relationship.

Carla: To wrap up, Karl, is there anything that I didn't ask you that you think it's important that you would like to share?

Karl: Probably, but I can not think of it right now! I really enjoyed this conversation, Carla. Thank you!

Carla: Karl, thank you very much for your time. Thank you very much!

## 4 Discussion

The conceptualization and clinical consideration of relational disquiet in family therapy can have profound implications for clinical practice, particularly for therapists who adopt a relationally informed approach. In reflecting upon potential uses, we identified three groups of practices that may assist therapists wanting to expand the generativity within relational disquiet: noticing, sharing, and acknowledging.

### 4.1 Noticing Relational Disquiet

The first practice involves embracing our ability as therapists to notice various forms of relational disquiet or disquieting experiences (Simão 2000) during the therapeutic process. Arguably, the ability to recognize relational pain is critical for therapy, as it is why people seek out family therapy services in the first place. While we would think all humans have the ability to notice pain, in our training at the CFTC we hope that therapists will intentionally grow this skill. Anecdotally, past interns at CFTC have consistently shared that befriending the notion of relational disquiet—collectively inviting and welcoming the *noticings* of relational disquiet as something worth doing, for instance, within clinical supervision and post‐session debriefing—has helped them develop their skill to identify various forms of relational pain. Noticing relational disquiet may include deliberately attending and responding to various forms of objectionable talk (Sametband et al. 2023; Strong and Tomm 2007): from noticing upfront objections between two people participating in the therapy session (e.g., “I disagree with you!”; “that's not true!”), to more subtle ways of protesting. For instance, people may perform an objection by becoming monologic, or less open to being influenced by others' words and deeds; or perhaps more prone to getting stuck on a certain view, or interrupting others in non‐generative ways; or perhaps they may start fidgeting in their chairs, or protest by frowning, displaying hesitance in their talk (“weeeell…”; “yes, but”, using disclaimers), or perhaps simply by remaining silent and withdrawing from the current conversation. By becoming more sensitive to recognizing relational disquiet within objectionable forms of talking and relating, and by treating such relational disquiet as calls to appropriate therapeutic action, therapists may open space for these relational disquiets to be shared and publicly acknowledged, which in turn may bring forth more generativity (we unpack the potential generativity of sharing relational disquiets later on).

During the interview, Karl described feeling and noticing disquiet when Colleen interrogated him about his professional responsibilities during their first session. It seems that he could sense and notice a subtle form of objection embedded in Colleen's question; as if there might be something “wrong” with it. In our view, by noticing the disquiet Karl tried to move forward with the disquiet by embracing self‐reflexivity. He seemed to relate to “his” (her?) disquiet as a form of pain; perhaps a signal of “feeling unsafe,” and tried to acknowledge and create room for Colleen to share her legitimate pain and perhaps reluctance to engage in therapy. At the same time, he noticed another source of disquiet: his concerns for Millie's safety. As Karl clearly defines, disquiet can be seen as a “bifurcation point. It could be the beginning of more trauma, injury and damage. Or, it could be the beginning of creativity and new developments that are therapeutic and healing.”

### 4.2 Sharing Relational Disquiet

Therapists may share their awareness of a relational disquiet by pausing to reflect, asking further questions, or sharing their perceptions of it. Therapists may share their own disquiet and ask whether somebody else might be noticing or experiencing the current situation as disquieting. Using circular questions, they can explore the experience of others in the room (e.g., how do you think this conversation is going for your mom right now?), or invite clients to name what their disquiet may be responding to (e.g., what is your mom's pain about?; what may be some positive and negative consequences of your mom sharing what her pain is about?; why would those be positive or negative consequences for you?). By asking questions animated by their own disquiet, therapists can help others in the room to talk (disquiets)‐into‐being, something we see as therapeutic inasmuch as it brings forth othered ways of relational experience as fully legitimate. In short, we view it as a potentially healing act of love.

In addition to posing circular questions, therapists at times share their disquiet more directly, either by self‐disclosing comments (May I share some disquiet I felt when I heard you sharing that?); or by *performing* disquiet, as shown by Karl's response to Colleen's request of not calling Child Protective Services. In that instance, we wonder if Karl was acting under the influence of his own disquiet as he started to recognize that Colleen's request could place him in a dilemmatic space, or a conundrum between two (perceived) obligations. On the one hand, reporting a violent incident and maximizing safety for those most vulnerable in the relationship; on the other hand, nurturing the therapeutic relationship through honoring Colleen's request while confirming that Millie was safe. While Karl did not explicitly share his disquiet with Colleen, he was able to perform his disquiet in recognizable ways. He explored further where Millie was at the moment and made sure that she was safe, asked whether Millie had been hurt during the incident, and perhaps demonstrated some relief (with his facial expression, with his tone of voice) when confirming that Millie would be staying with her maternal grandmother. By asking all these questions with a certain level of concern, we imagine Colleen may have recognized/felt that Karl was in disquiet about her daughter's safety and was examining the level of risk and safety measures in place. He even asked if Colleen's mother would be willing to be involved in the therapeutic process and was transparent about his need to get collateral information. As an element of generativity that we view ensuing from this (performed) concern after Colleen's request, we think that Karl's *disquiet‐sharing* may have influenced or even occasioned Colleen's willingness to clarify her daughter's current safety state and possibly a sense of relief from her initial disquiet. Moreover, this conversation may have generated a shared sense of relief that seemed to influence Karl's accepting Colleen's request to not call Child Protection Services at that time.

### 4.3 Acknowledging Relational Disquiet

The practice of acknowledging relational disquiet involves legitimizing the relational disquiet by bringing it into the open or ac‐knowledge it. The prefix *ac* in the word acknowledgment is an adaptation of the Latin word *ad*, which means to, towards, or added to. Acknowledgement is an explicit expression of something already known (by the speaker at least) which is communicated to another person, and sometimes to a whole community (Tomm and Govier 2007). This could involve reflective listening exercises (Tomm and Acton 2011) where someone is invited to mirror back in words a form of pain, and thus participate in the co‐construction of (mutually recognizable) influences generating disquiet in an individual (e.g., what do you hear your mom saying when she shares her disquiet?). Or perhaps this could be used to reflexively co‐construct new self‐understandings as a person, as a family, or as a community, which we see as key to fostering therapeutic change (see e.g., Gaete et al. 2020). For instance, asking: “what does this disquiet tell you about what is important for your mom?” could potentially lead to articulating common goals for therapy by incorporating the responses to a common agenda (e.g., what is good for you about what is important to your mom? Is this important to you as well? Is it important for our work in therapy? How so?).

Although we cannot illustrate from the interview what actions Karl and Colleen engaged in to acknowledge their disquiets, we imagine that Karl and Colleen brought forth a shared understanding of their concerns as legitimate and warranted, and requiring further discussion. In other words, safety is very important and a hearable concern in the context of therapy, both in relation to confidentiality practices and protecting/respecting the dignity of all people, particularly those who are most vulnerable in a therapeutic relationship. Further, we would think that the disquiet gained power (in its therapeutic potential) when it was legitimized (i.e., acknowledged) by Karl's professional team, which supported him in his decision‐making process.

As a final remark, we invite readers to consider Karl's suggestion about understanding relational disquiet as an embodied phenomenon. Such embodiment acknowledges its situated complexity in all its facets: it is a form of *feeling*, involves a form of *judgment* (implicit “wrongness”), and a *cognition* in the sense that the feeling is *about* something the feeler cares about (*values*, relational hopes) within a lived situation. Our hope is that co‐explorations centered on noticing, sharing, and acknowledging relational disquiets may assist those navigating such disquiets to better understand their circumstances in ways that help them realize their relational hopes.

## Conflicts of Interest

The authors declare no conflicts of interest.
